# Unpaid dementia carers: a scoping review of the caregiving trajectory and changing short break needs

**DOI:** 10.3389/frhs.2025.1583975

**Published:** 2025-10-24

**Authors:** Maria Caulfield, Diane Seddon

**Affiliations:** 1 Applied Dementia Studies, School of Nursing, Healthcare Leadership and Public Health, Faculty of Health and Social Care, University of Bradford, Bradford, West Yorkshire, United Kingdom; 2 Ageing & Dementia @ Bangor/Dementia Services Development Centre (DSDC) Wales Research Centre, School of Health Sciences, Bangor University, Gwynedd, Wales, United Kingdom

**Keywords:** caregiving dynamics, caregiving career, dementia, short breaks, respite, support planning

## Abstract

**Introduction:**

Short breaks are essential to enable unpaid carers to have a life alongside caregiving. However, there is limited understanding of how carers' break needs evolve over time. This scoping review aimed to identify models of dementia caregiving to explore how short break needs may change across the caregiving career.

**Methods:**

The review followed the best practice guidelines by and Arksey and O'Malley and Levac et al. A search was conducted across four databases in 2023 and rerun in 2025: Applied Social Sciences Index and Abstracts, CINAHL, MEDLINE, and APA PsycINFO.

**Results:**

Eleven models were identified, outlining various stages of caregiving. These models focused specifically on spousal carers or predominantly included spouses. They demonstrate that caregiving is a dynamic process, marked by increasing demands on carers' time and shifting relational dynamics. The models suggest that short break needs may change in response to certain circumstances, with a shift in emphasis from relational well-being in the early stages to rest and recuperation in later stages.

**Discussion:**

The findings highlight the importance of regular practitioner engagement to monitor and discuss changing break needs, and the need for accessible community and social opportunities that support a mutual respite experience for both the carer and the person with dementia. Key knowledge gaps are identified, including the potential role of short breaks in supporting the person with dementia during the transition to residential care, and in helping carers adjust to this new phase of caregiving. Future research could also examine the best ways to capture and monitor short break needs over time, including during Carers' Assessments and other short break conversations.

## 1 Introduction

As the global population ages, and the number of people living with dementia increases, most of the long-term care and support is provided by family members and friends (1, 2). Referred to as “unpaid carers”, the care they provide is extensive, can last for many years, and can increase in intensity over time (3). For the estimated 55.2 million people with dementia worldwide, the economic equivalent of unpaid care is valued at approximately 651 billion USD, representing half the total global economic burden of dementia, estimated at 1,313 billion USD (4).

While caregiving, with appropriate support and resources, can be a fulfilling experience, bringing opportunities for growth and satisfaction (5, 6), adverse effects on the carers' health and well-being are widely reported (7, 93). The effects of providing care significantly impact on many domains of life, making caregiving a social determinant of health (8). Particularly for female carers and who provide high intensity of care, these responsibilities exacerbate existing inequalities (9).

Systematic reviews underscore the multiplicity of carer support needs. These needs encompass carers' personal self-care, including sleep, social engagement, and emotional support (10). Additionally, carers require advice and assistance from professionals as well as practical support from services to help them provide care for the person with dementia (11, 12). A range of interventions exist to support carers in their caring role (13). Among these, there is consistently strong evidence supporting the need for short breaks, which provide time away from caring routines and responsibilities (14–16).

The term 'short break' encompasses a wide range of services and activities, ranging from in-home support by care staff, to day care centers, residential stays, supported holidays, and community-based activities. Breaks can be planned regularly on a weekly or monthly basis or taken as needed. They may last for a few hours during the day or extend over longer periods. Breaks can occur during the day, for example, to allow carers time to meet friends or engage in a hobby, or overnight, to enable them to get a full night's rest. They may also involve time spent apart or together, if preferred, so that both the carer and the person with dementia can enjoy time together in a new environment outside of their usual routine. Shared Care Scotland (17) highlights the key features that define a short break:

A short break is any form of service or assistance, which enables the carer(s) to have sufficient and regular periods away from their caring routines or responsibilities, with the purpose of supporting the caring relationship and promoting the health and well-being of the carer, the supported person and other family members affected by the caring situation.

Short breaks can facilitate a life alongside caring (18–20), which is a policy priority in the United Kingdom (UK) and internationally. In Wales, supporting a life alongside caring through the provision of short breaks is one of four national priorities for carers. The Welsh Government states that all unpaid carers must have the opportunity to take breaks from their caring role to enable them to maintain their well-being (21). Similarly, the International Alliance of Carer Organisations recognises carers' right to “time off” from caring to maintain their physical and mental health as one of six universal priorities (22). To translate this policy priority into practice, improving access to short breaks is a core component of global strategies for carers, including *Enabling Carers to Care: An EU Strategy to Support and Empower Informal Carers* (23), Ireland's *National Carers' Strategy* (24) and the Canadian *Caregiver Strategy* (25).

Due to its evolving nature, dementia caregiving has been conceptualised as a “caregiving career” (26). Carers navigate through different stages of caregiving, characterised by events such as recognising the necessity of caregiving, undertaking tasks associated with care either at home or in a care home, and ultimately relinquishing the caregiving role (26), each presenting unique stressors and impacting the depletion or utilisation of coping resources (27, 28). Understanding caregiving as a dynamic process encourages thinking around how interventions and support can be adapted as carers transition through different stages (29).

Although a substantial body of evidence highlights the dynamic and complex trajectory of caregiving and its impact (94, 95), limited attention has been paid to understanding carers' evolving need for short breaks and how the nature of these breaks may change as they progress through their caregiving careers. This gap in knowledge is highlighted in a scoping review of the literature on short breaks, mapping the evidence pertinent to carers for older adults, including those caring for people with dementia (30). The review highlights several gaps in knowledge, notably in understanding how the needs, preferences, and desired outcomes for short breaks may evolve over time for carers and those they support. This includes considerations such as shifts in preferred settings and activities, variations in the need for breaks to be taken alone vs. breaks together with the person with support needs, and the optimal duration and type of short break for achieving positive outcomes such as an improved sense of satisfaction in caregiving and greater choices about caregiving, including limits in caregiving capacity and willingness to provide care (30).

This scoping review aimed to identify models of dementia caregiving to explore whether they can offer insight into carers' short breaks needs across the caregiving career.

## 2 Method

### 2.1 Search strategy

The scoping review followed guidelines by Arksey and O'Malley (31) and the refinements by Levac et al. (32). Reporting followed the Preferred Reporting Items for Systematic Reviews and Meta-analyses extension for scoping review (PRISMA-ScR) (33).

An initial search in Medline (EBSCO) identified articles using the key words “caregiving career” and “dementia”. The text words found in the titles and abstracts of pertinent articles were used to construct a full search strategy. A subject librarian helped develop the search strategy. The search strategy was piloted in Medline (EBSCO) and APA PsycINFO (EBSCO). The search terms are shown in Table 1.

**Table 1:** Search terms.

Search terms
“caregiving process” or “caregiving trajectory” or “caregiving model” or “caregiving stages” or “caregiving phases” or “caregiving career” or “multidimensional model of caregiving” or “changes in caregiving over time” or “longitudinal changes” or “temporal model* of caregiving” AND dement* or Alzheimer* OR Lewy OR Fronto* OR cognitiv* impair*

### 2.2 Study identification

The search was initially conducted in January 2023 as part of a PhD project and rerun in July 2025. Four health sciences databases were searched: Applied Social Sciences Index and Abstracts (ASSIA)(ProQuest); CINAHLPlus (Cumulative Index to Nursing and Allied Health Literature: EBSCO); MEDLINE (EBSCO), and APA PsycINFO (ProQuest). The publication date was set to start from 1986, reflecting the earliest publication by Chenoweth and Spencer (34) identifying stages in a caregiving career.

### 2.3 Study screening

Studies were included if they identified and named specific stages of the dementia caregiving career and were written in English. Models of dementia caregiving were defined by their identification and naming of stages along the caregiving career. To determine a career ’stage' the Aneshensel et al. (26) definition was used:

A heuristic device that helps detect the threads connecting each part of caregiving to its other parts and identify conditions that move caregivers along their career trajectories at different rates and at different psychological and material cost to themselves.

To avoid subjective interpretation of stages, studies were excluded if they did not define specific stages of the dementia caregiving career. For example, studies that provided only a temporal description of changes in experience, needs, support preferences (35, 36) or interaction with formal services over time (28), were excluded if they did not clearly outline distinct stages. Grey literature was not included in the search, as the primary focus was to include sources that have undergone a peer-review process to support the reliability and validity of the findings. Consistent with scoping review methodology, no quality appraisal was conducted (31, 32).

### 2.4 Study selection

**Figure 1:**
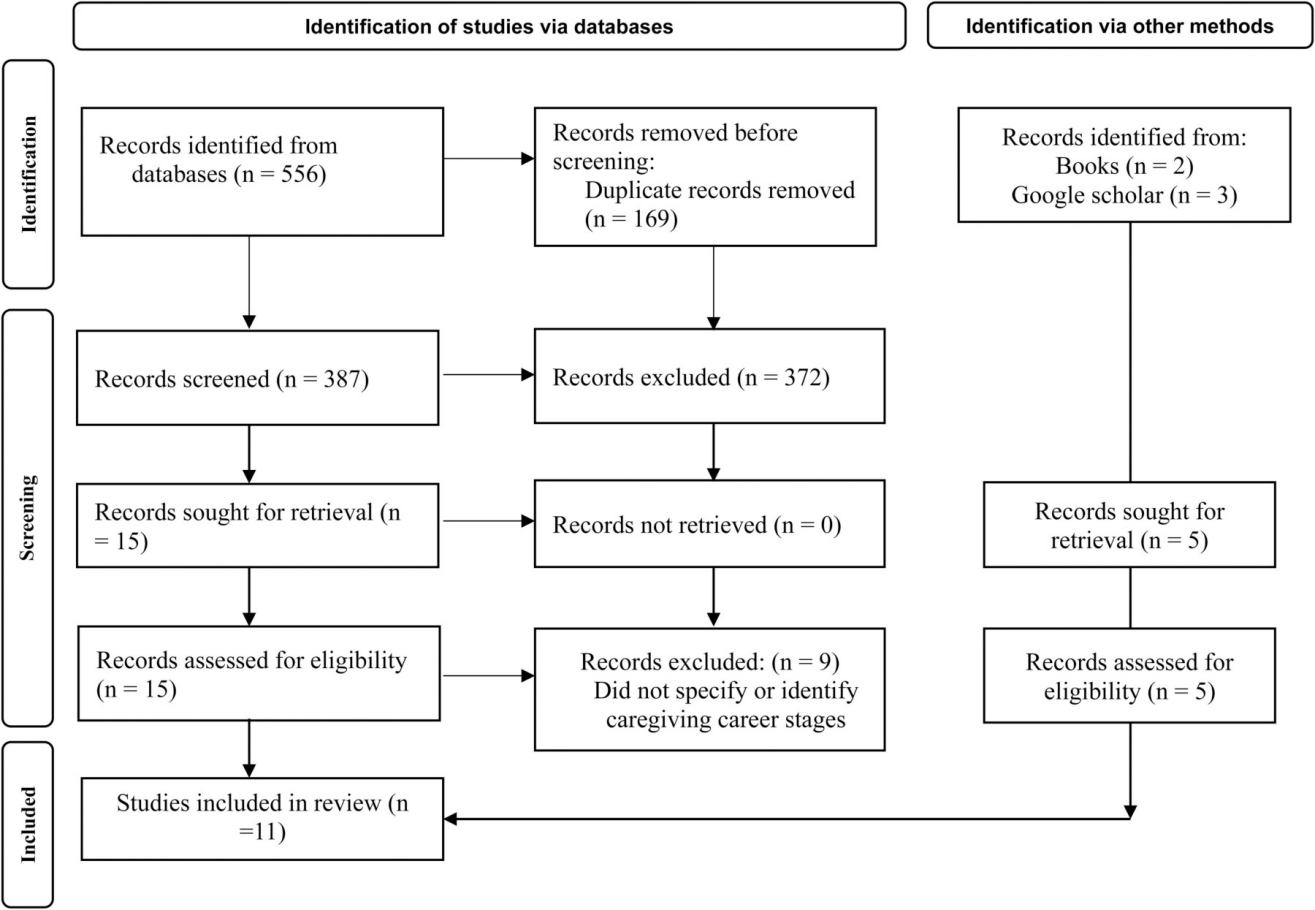
PRISMA flowchart.

The search generated a total of 556 articles. Two books by Aneshensel et al. (26) and (37) were included as additional sources. The search results were exported to Mendeley reference manager and duplicates removed. Titles and abstracts were screened by the first author and potentially relevant articles were screened in full, assessed against the eligibility criteria, and discussed with the second author. Citation tracking (forward searching) and reference screening (backwards searching) of included full-text articles were conducted. The PRISMA flowchart shows the article selection and screening process; see Figure 1.

### 2.5 Charting the data

Data and study characteristics from articles that met the inclusion criteria following full-text screening were extracted into an Excel spreadsheet by a single reviewer (MC). The data extracted included the author, year, research location, title, study aim, sample characteristics, methods, and the number of caregiving stages.

### 2.6 Data synthesis

The synthesis of the eleven models was guided by Byrne's (38) reflexive approach to qualitative data analysis. The aim was to identify commonalities in how the models' trajectories, with particular attention to distinctive features of the dementia caregiving experience and to key junctures where support needs may shift in nature or intensity. Analysis began with familiarisation with the textual descriptions of each model, engaging with how they constructed and represented the dementia caregiving experience, and noting the moments where carers' needs appeared to shift or intensify. This was followed by visually plotting the stages of each model to enable comparison in how the caregiving career was structured in terms of the number and sequence of stages, with an initial clustering of stages into the beginning, middle, or end of the caregiving career. The descriptions of each stage were then examined to inform interpretive reflections on the challenges and opportunities they represented, as well as potential insights into carers' evolving needs for short breaks at different points in their careers. Through this process, the stages were refined into five distinct phases that captured notable differences across models. The findings are presented as an interpretive synthesis of shared trajectories and key transitions within the caregiving career, with emphasis on how short breaks might best meet carers' needs at each stage.

## 3 Findings

### 3.1 Model characteristics

The review included eleven studies describing models of dementia caregiving. The study characteristics are presented in Table 2. The studies were published in the USA (4), UK (3), and Canada (2), China (1), and Japan (1), between 1986 and 2022. Three studies were published in the 2000s, six in the 1990s, and two in the late 1980s.

**Table 2:** Characteristics of included studies.

Author, year, research location, and title	Aim	Participant characteristics	Methods	Number of caregiving stages identified
Tu, 2022 China The trajectory of family caregiving for older adults with dementia: difficulties and challenges	To investigate the trajectory of family carers’ struggles from home care to a care institution	**13 family carers:** 5 spouses (4 wives and 1 husband) 8 adult children (6 daughters and 2 sons) Mean age: 63 yrs Duration of providing care: < 1 month to 5 yrs Ethnicity: Chinese	Ethnographic The researcher spent 14 months in the care institution, observing interactions between staff, residents, and families. During visits, family members were interviewed about their long-term caregiving experiences, both at home and within the facility	2 overarching stages 4 tipping points, triggering the decision for institution placement
Cooper, 2022 USA “That's when the relationship shifted”: Relational and communicative turning points in Alzheimer's disease and related dementias	To investigate the specific relational turning points experienced by spousal carers across the trajectory of dementia.	**18 spousal carers** (10 wives and 8 husbands). Mean age: 69 yrs. Mean years married: 38 yrs Mean disease length: 5 yrs Race: White: 13 Black: 2 Multiracial: 2 Hispanic: 1	Retrospective interviews explored spousal carers’ relational experiences throughout the progression of the disease Participants created turning point timelines to identify key moments in their relationship	3 overarching stages encompassing 9 discrete relational turning points
Kokorelias, 2020 Canada A grounded theory study to identify caregiving phases and support needs across the Alzheimer's disease trajectory	To develop a conceptual framework of caregiving phases across Alzheimer's disease and caregiving trajectories	**40 family carers:** 20 spouses (10 wives) 20 adult children (10 daughters and 10 sons) Age range: 45 to 88 yrs	Grounded theory. Semi-structed interviews collected data on the experiences and responsibilities of carers across the disease trajectory	5 caregiving stages.
Pfeiffer, 1999 USA Stages of caregiving	To describe the stages of caregiving as experienced by carers, the issues they faced, appropriate services for that stage	No characteristics reported	Long-term observation of family carers for people with Alzheimer's disease. The exact duration is unspecified	7 caregiving stages
Nolan, 1996 UK Understanding Family Care: A Multidimensional Model of Caring and Coping	To outline a temporal model of the dementia caregiving process based on the longitudinal study of dementia carers	**58 family carers:** A mixture of carers new to their role; experienced carers who had been providing care for many years; carers who had placed the person with dementia in care/nursing home or the person with dementia had died	In-depth interviews conducted over a three-year period.	6 caregiving stages
Aneshensel, 1995 USA Profiles in caregiving. The unexpected Career	To highlight the course of caregiving for people with dementia. To capture the transitions and changing conditions that carers experience during the trajectories of their caregiving activities	**555 family carers** (at baseline) Relationship with person with dementia (%): Wife: 34.2 Husband: 24.5 Daughter: 31.2 Daughter in Law: 3.2 Son: 6.7 Son in Law: 0.2 Spousal carers age (%): Less than 65: 22.8 65–74: 45.5 75 and older: 31.7 Race (%): Non-Hispanic white: 83.8 African American: 10.6 Hispanic: 3.1. Asian American and other: 2.5	Four-in-person semi-structured interviews conducted at one-year intervals: baseline, caring at home, institutional care, and bereavement	3 caregiving stages
Wuest, 1994 Canada Becoming strangers: the changing family caregiving relationship in Alzheimer's disease	To explore the reciprocal process of “becoming strangers”	**15 family carers:** 8 spouses (5 wives) 5 daughters 1 son 1 sister Age range: 28 to 83 yrs.	Grounded theory. During data collection, emerging commonalities and relationships within the data informed subsequent observations and interviews, helping to clarify developing themes	The continuum of ‘becoming strangers’ encompassed 3 stages
Kobayashi, 1993 Japan Developmental process: family caregivers of demented Japanese	To identify how carers: a) perceived the person with dementia and their attitudes toward them, b) how these perceptions and attitudes changed over time, c) when and how these changes occurred, and d) the specific changes they displayed	**49 family carers:** 19 daughters-in-law 11 wives 10 daughters 8 husbands 1 son Mean age: 57.9 yrs. Ethnicity: Japanese	Qualitative descriptive. Semi-structured interviews explored carers’ understanding and perspectives of the person with dementia	7 stages during which the carer demonstrated perceptions and attitudes toward the person with dementia
Willoughby and Keating, 1991 UK Being in control: the process of caring for a relative with Alzheimer's disease	To understand the process of dementia caregiving from the perspective of family carers	**10 family carers** (from 7 families): 3 wives 4 daughters 3 sons Range of yrs providing care: 1.5 to 15	Using a grounded theory approach, participants took part in two unstructured interviews. The first focused on early changes in the person with dementia, the diagnostic process, decisions around care placement, interactions with paid carers, and sources of support. The second explored caregiving trajectories, including when caregiving began, expectations regarding its end, and how family dynamics evolved over time. The researcher also conducted observations of carers during visits to their relatives in the care home	5 caregiving stages
Wilson, 1989 UK Family caregivers: the experience of Alzheimer's disease	To conceptualise the course of Alzheimer's disease as experienced by family carers	**20 spousal carers** (14 wives and 6 husbands). Marital status: 18 married 2 widowed Age range: 29 to 85 yrs Mean age: 62yrs Ethnicity: Asian: 1 Native American: 2 White: 17	Semi-structured in-person interviews explored problem recognition, the diagnostic process, and progressive decline Thematic analysis identified themes reflecting carers’ lived experiences throughout the course of dementia	8 caregiving stages
Chenoweth and Spencer, 1986 USA Dementia: The experience of family caregivers	To explore family carers’ experiences from the recognition of dementia symptoms through the progression of the disease	**289 family carers** (%): Wives: 41 Husbands: 14 Not specified: 45 Race (%): White: 99 Other: 1 Age (yrs) (%): 24 to 40: 24 41 to 60: 39 61 to 82: 37 Religious preference (%): Protestant: 60 Catholic: 26 Jewish:4 None or other: 10	Data were collected through a non-validated questionnaire, supplemented by photographs, medical records, and letters. In addition, 13 carers participated in telephone interviews to provide more detailed accounts of their experiences	4 caregiving stages

The methodological approach was reported in four studies. Three studies employed grounded theory (41, 43, 45) and one study used ethnography (39). In-depth semi-structured interviews were the main method of data collection for all studies. These were either conducted at a single point in time (39–41, 44, 46), or repeated over a longer period, ranging from one year to three years (26, 37, 45). In addition to interviews, questionnaires were used by Chenoweth and Spencer (34) and Cooper et al. (40) asked participants to draw timelines to identify “turning points” across the disease trajectory. All studies included spousal carers, and adult children were included in the sample of six studies (26, 39, 41, 43–45).

**Figure 2:**
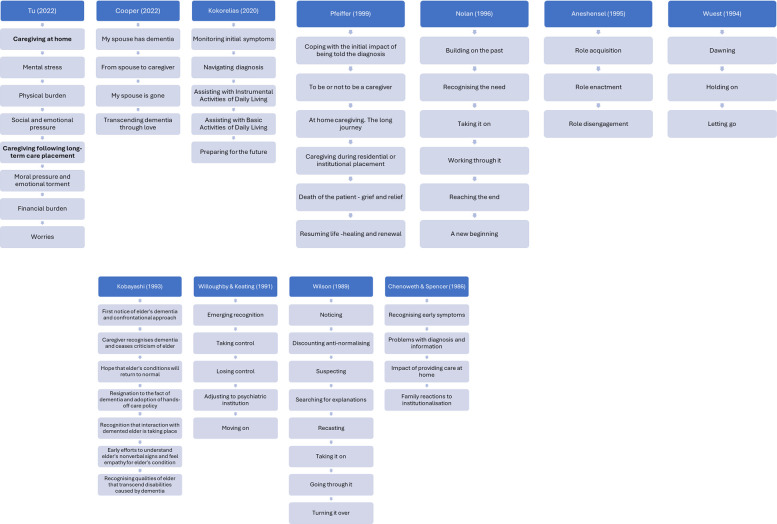
Visual schematic of the titles given to each caregiving stage.

A schematic of the caregiving stages from each study is presented in Figure 2. The models progress in a sequential manner and share several common features in the progression of stages: beginning with the recognition of dementia symptoms, followed by diagnosis, acceptance of the need for care and support, adaptation to and management of dementia, balancing caregiving responsibilities, transition to residential care, and culminating in rebuilding life following the death of the person with dementia. While dementia symptoms may manifest and be experienced in different ways, the models tend to follow a pattern of escalating carer's responsibilities and growing dependency as dementia advances.

The transition between stages is typically demarcated by an event, such as receiving a diagnosis, accepting formal support, or the person with dementia moving permanently into a care home. In the study by Willoughby and Keating (45), movement between stages is determined by a cognitive shift, defined as “a change in thinking, a new understanding, and new insights into an experience” (45). All models depict stages that reflect the diverse challenges faced by carers, including practical caregiving tasks and various sources of stress. These stressors may arise from the condition itself, such as the level of cognitive impairment, behavioural changes, or difficulties with activities of daily living, as well as from carers’ subjective experiences, including feelings of role overload, or losses related to self and to the caregiving relationship. In some models, stages are more pronounced by their orientation towards caregiving tasks (34, 39, 41, 42), characterised as the “activities, tasks, and focus of care contributed by carers to assist the person with dementia” (41). Three models focus on the relational and emotional shifts that occur over time such as changes in intimacy, communication, and reciprocity (40, 43, 44).

### 3.2 How do the models inform our understanding of changing short breaks needs

#### 3.2.1 Recognition of dementia symptoms

The beginning stage for many models describes the “dawning” (42) or “emerging” (45) realisation that something is wrong, often accompanied by anxiety and uncertainty. Aneshensel et al. (26), describes entry into this stage as having a nebulous quality, owing to the insidious onset of dementia. Carers often struggled to pinpoint the initial changes, as symptoms often manifested through subtle personality or cognitive shifts or difficulties at work. As a result, models describe stages as “monitoring initial symptoms” (44), “noticing” (46), and “recognising early symptoms” (34). Carers oscillated between convincing themselves symptoms such as forgetfulness or changes in personality and behaviour were part of “normal” ageing and suspecting something more serious was underlying these symptoms (40, 41).

#### 3.2.2 Receiving a dementia diagnosis and acceptance of the need for care and support

Having identified troubling symptoms, carers embark on a “diagnostic quest”. Kokorelias et al. (41) characterise this process as “navigating diagnosis”, while Wilson (46) describe it as 'searching for explanations', and Chenoweth and Spencer (34) describe it as “problems with diagnosis and information”. The confirmation of a diagnosis is a significant juncture in the caregiving relationship. For some spousal carers, it brings relief as they can attribute a cause to behaviour, and there is an opportunity for honest communication (40). For others, the acknowledgment that their family member is embarking on a path of dependency leads to uncertainty regarding their own capacity, willingness, and ability to “take on” the caring responsibilities (37) or “to be or not to be …. a caregiver” (42). At this point in the caregiving career, depending on how advanced dementia is at the time of diagnosis, carers may not experience an acute need for breaks apart. Carers may have limited practical caregiving responsibilities in the early stages, however, the period following a diagnosis can be highly stressful as couples adjust to its emotional and relational impact (26). It may be that taking breaks with the person with dementia can help to maintain or strengthen a couple's sense of shared identity and support the health and quality of the relationship (17, 47). Equally, breaks that incorporate an element of peer support may be particularly valuable during this stage of caregiving, helping couples adjust to the diagnosis while learning from and connecting with others in similar situations (48, 49). These early stages in the caregiving career are a fertile ground for introducing the notion of short breaks and recognising their potential benefits, both now and in the future.

#### 3.2.3 Providing care at home: adapting to dementia and balancing care responsibilities with carer wellbeing

Within the longest and most demanding phase of caregiving, various challenges persist, termed as “working through it” (37), “going through it” (46), “holding on” (43), and the “long journey” (42). These stages represent the caregiving experience at home, marking the carer's transition from supporting instrumental activities of daily living (e.g., cleaning, cooking) to more complex personal care tasks (e.g., bathing, toileting, emotional support, financial management etc.) While carers may have a good understanding of dementia, they are responding to emergent problems and situations on a trial-and-error basis (37, 44, 46), often with one source of stress replacing another (26). Sleep deprivation was common and left carers feeling debilitated to deal with their daily responsibilities (34, 39). Carers sought balance in their life, but as the caregiving demands became more intensive, carers had less time or opportunity to sustain friendships or pursue interests, leaving carers feeling isolated and alone (34, 39, 43).

We suggest that the need for short breaks becomes particularly acute during the stage of caregiving at home, when increasing dependency places greater demands on the carer's time and attention, and can deplete their coping resources. Many models suggest a growing sense of confinement, as carers feel trapped or overwhelmed by their responsibilities (26, 34, 39, 42). It is during this stage where needs and preferences for short breaks become more personalised, and where it is most likely that some form of professional intervention is needed to support the realisation of more regular breaks, that can enable the carer to have time to themselves. To facilitate this, alternative care arrangements for the person with dementia are required (37).

Community-based initiatives that provide meaningful activities for people with dementia, while also offering carers a valuable short break, are highly valued (47, 50). Innovative models, particularly those emphasising nature-based activities, known as Green Care Farms, have been pioneered in countries such as the Netherlands and Norway, and typically involve extended periods outdoors and include health-promoting, tailored tasks such as animal care, gardening, crafts, woodworking, and food preparation (51, 52). Comparative studies have found that attendees of care farms report significantly higher emotional well-being, greater physical activity, and more frequent social engagement compared to those attending traditional care facilities (53–55). Additionally, family carers reported that their relatives with dementia slept better following visits to the care farm, which in turn contributed to improved sleep for themselves (56).

However, for individuals with dementia whose health is poor, or mobility is limited, or simply prefer it, remaining in the comfort and familiarity of their own home may be preferable. In such cases, in-home respite care can offer a valuable alternative, enabling carers to take breaks while ensuring the person with dementia remains in a familiar and supportive environment (57–59). This option is often preferred over others such as day care, which can present challenges when transportation is required, there are limited or inflexible opening hours, or when day care staff struggle to manage behaviours perceived as challenging or to support individuals who require assistance with toileting (60). Zhang et al. (61) analysis of in-home respite care illustrates how this form of short break support has developed to provide more tailored and specialised assistance for both carers and people living with dementia. Initially, in-home respite services primarily focused on basic household and caregiving tasks. However, over the past two decades, these services have evolved to include more complex care activities, such as administering medication, developing rehabilitation plans, and teaching caregiving skills, tasks that typically require trained medical, healthcare, social care professionals. Zhang et al. (61) also highlights that, in addition to offering domestic support, in-home respite care has increasingly addressed the emotional well-being of family carers by incorporating stress-relief interventions and psychosocial support.

While in-home respite can be a valued form of support, this form of break may be less effective in preparing couples for the potential next step in the caregiving career compared to other respite services. For example, day care services or short stays in residential care are often seen as a transitional step toward residential or nursing home admission by offering trial periods of separation and gradual adjustment to new care environments (47, 62). However, carers have raised concerns around the quality of residential care, citing lack of personalised care and activities, inadequate staff training in specialised dementia care, long waiting lists, and fear that the person with dementia will return home in a more distressed state (62–65).

#### 3.2.4 Transitioning to residential care

All models refer to the stage when carers came to recognise that continuing to provide care at home is no longer in the best interests of either themselves or the person with dementia (37). This shift toward care home placement resulted from a gradual accumulation of events rather than a single defining moment. Contributing factors included the perception of increasingly aggressive behaviour by the person with dementia, a breakdown in the relationship to the extent that they felt like strangers, or carers feeling trapped in their caregiving role (26, 39, 40, 43). Upon this transition, the locus of caregiving responsibilities shift. Carers redefine their caring role, with many supplementing the care provided by care staff by often engaging in tasks such as preparing home-cooked meals and by spending quality time with the person with dementia (26). While physical burden may lessen, some carers encounter new “moral and emotional torment” (39), along with financial strains arising from the significant costs associated with accommodation and care (26, 42). Concerns about the quality of care often prompt carers to make frequent visits to the care home, where logistically possible (39). While it might be assumed that carers' needs for break lessen once the person with dementia moves into residential care, models suggest the emotional and physical consequence of caregiving remain substantial. At this stage, short breaks that incorporate elements of emotional support or therapy, such as counseling or relaxation, may help carers maintain their well-being and adapt to caregiving in a new setting and respond to new challenges.

#### 3.2.5 Rebuilding life following the death of the person with dementia

The final stage, reported in four models, relates to the rebuilding of life following the physical passing of the person with dementia (26, 37, 42, 45). While physical death marks a discrete event, disengagement from the caring role is a far more gradual and intricate process (26). Feelings of pre-death grief, referred to as anticipatory grief (66), may have already occurred during the caregiving career due to the compounded serial losses in the dementia process. However, carers must navigate the additional stage of “role disengagement” after the physical passing. This process involves bereavement, recovery, and social reintegration (26). As a part of their healing process, some carers may choose to be active in supporting other carers through their career, through volunteering and mentoring (26, 42). At this stage, it becomes evident why short breaks throughout the caregiving career are essential for maintaining social connections and preserving a sense of identity beyond the caregiving role. It can be postulated that a carer's healthy adjustment following the death of the person with dementia may, to some extent, depend on their ability to maintain a life alongside their caregiving responsibilities.

## 4 Discussion

This study identified eleven models of dementia caregiving to explore the insights they offer into carers' evolving needs for short breaks. While the specific nature of a break activity may vary across different stages, fundamental principles, such as mutuality, continuity of quality of care, and flexibility, can be identified as essential components. It can further be postulated that, in line with the trajectory of dementia caregiving and tendency to demand more on carers time, there is a gradual shift in the types of short breaks that carers prioritise. In the early stages, the focus is more on maintaining relational and individual well-being, with breaks that support both partners' skills, interests, and capabilities. However, as caring responsibilities increase, there is a growing need to prioritise breaks that allow carers to rest and recuperate, ultimately supporting their capacity to continue to care. More frequent or longer breaks may be necessary, and these may involve the provision of alternative care by skilled care professionals who can support people with dementia to engage in meaningful activities or manage complex care tasks.

The models emphasise the dynamic and shifting nature of caregiving, shaped primarily by the progressive course of dementia and the increasing demands of providing care. While the models outline broad stages that help chart the typical trajectory of caregiving and highlight common patterns, there remains significant variation within each stage. Factors unique to each caregiving situation, such as the carer's own health, the quality of the relationship prior to the onset of dementia, the strength of the support network, family responsibilities, employment, and financial resources, play a critical role in shaping the caregiving experience (67–69). This has important implications for how short break needs are discussed and monitored, and how short break options are designed and delivered.

The likely changing nature of carer short break needs underlines the importance of the regular practitioner engagement to assess, monitor, and review those needs. Proactive engagement is vital because, as caregiving progresses, carers may require encouragement to recognise or accept the need for a break, and support to identify and plan for appropriate breaks (30, 70). In the UK, Carers' Assessments are often, although not exclusively, the standard route for identifying short break needs and supporting the planning of breaks. However, the legitimacy and value of the assessment process is poorly perceived by both carers and practitioners (71, 72). According to the 2022 State of Caring Wales report, a third of carers (33%) who received an assessment felt that their need for regular breaks was not meaningfully considered. Service evaluations in Wales (73, 74) and in Scotland (75) also highlight considerable variation in how Carers' Assessments are conducted, in terms of format and content. A range of tools and resources have been considered to support more meaningful and skilled conversations about short breaks. These include the potential use of images to enhance short break conversations (76), short break toolkits that assist carers and practitioners in thinking through and organising short breaks (77, 78), and an online intervention to help carers schedule and plan their break time to maximize its benefit (79).

At any stage in the caregiving career, for a break to be meaningful for carers, the evidence highlights the importance of mutuality in experience and outcomes (70, 80). To psychologically 'switch off' from their caring role, carers must trust that the person they support is receiving high-quality care in their absence (81, 82). It is particularly important that a person with dementia is offered opportunities to engage in meaningful activities that reflect their interests and maintain their skills and abilities (83–85). Carers need reassurance and confidence in the competence of care staff and the overall quality of care being provided (81). This evidence supports the need for a dyadic approach to short break planning and service delivery (80). Shared Lives is one example of short break service founded on the building a good triadic caring relationship between the carer, person with dementia and care staff (86). The service has demonstrated several meaningful outcomes for both carers and those they support. These outcomes are supported by continuity of care from familiar staff, which fosters trusting, long-term relationships, shared choice and control over how and when breaks are taken, and flexible provision that adapts to changing needs over time.

This review highlights important knowledge gaps in our understanding of potential for short break at different stages of the caregiving career. One emerging area of interest is how short breaks might act as steppingstones to facilitate a gradual transition to a new care environment (87, 88). Such an approach could help both the person with dementia and the carer adjust to separation and time apart, more positively by allowing them to become familiar with the care home setting and its staff (47), at what is often a distressing time for the couple (89). However, the feasibility of this approach depends on several factors, including the health and mobility of the person with dementia and the care home's capacity to support experiences or phased admissions. In practice, limited bed availability, staffing constraints, and additional costs may make this difficult to implement. The findings also highlight the ongoing responsibilities of caregiving even after a person with dementia moves into a residential care home and corroborates previous research showing that this is a stage where the need for breaks remains high but is often unrecognised (17, 47). How breaks at this stage can support carer well-being and adjustment remains an under-researched area but may be critically important in helping carers adapt to a new stage of life.

### 4.1 Limitations

The models identified in this review offer a general overview of the stages that characterise the caregiving career. While they outline a trajectory of increasing demands on carers, the variation within each stage and the nuances that define individual caregiving relationships and situations are impossible to fully capture. As such, it is difficult to provide precise examples of what short breaks may look like at each stage.

There is also a considerable time gap between the publication of studies. In part, this may be due to the search strategy that excluded studies that did not explicitly identify stages in the caregiving career. Longitudinal research has revealed changes in caregiver burden (90), subjective stressors (18), and appraisal of the caregiving role over time (91). Thus, the examination of longitudinal studies in dementia caregiving could provide additional insights into short breaks as circumstances evolve and is a key direction for future research.

A limitation of these models is their assumption of a gradual transition into the caregiving role. However, the time to receive a dementia diagnosis in the UK, estimated at approximately 18 weeks (92), can leave both carers and people with dementia without adequate support or understanding for extended periods. As a result, carers may already be experiencing significant strain by the time they become eligible for an assessment of support services. This may increase the urgency for respite and influence the type of breaks that carers require.

## 5 Conclusion

The models demonstrated that dementia caregiving is a dynamic process, marked by shifts in relationships, responsibilities, and transitions between care settings. The models suggest that short breaks can play a valuable role in supporting carers at all stages, helping them to maintain their well-being and capacity in their caring role. However, for breaks to serve as a form of preventative support, it is crucial that carers’ needs for breaks are discussed regularly to enable appropriate planning. For those designing and commissioning short breaks, the findings highlight the need for a range of accessible community and social opportunities that support a mutual experience of respite for both carers and those they support. These opportunities should ideally begin in the early stages of caregiving and extend to specialist short break services delivered by skilled care professionals in the later stages.
